# Demographics, presentation, and clinical outcomes after traumatic bifrontal contusions: a systematic review

**DOI:** 10.1007/s10143-019-01098-0

**Published:** 2019-05-16

**Authors:** N. Van de Zande, S. Manivannan, F. Sharouf, D. Shastin, M. Abdulla, P. D. Chumas, Malik Zaben

**Affiliations:** 1 Isala Hospital, Zwolle, Netherlands; 2 Department of Neurosurgery, University Hospital of Wales, Room 4FT 80E, 4th Floor, Heath Park, Cardiff, Cardiff, CF14 4XN UK; 3 Neuroscience and Mental Health Research Institute (NMHRI), School of Medicine, Cardiff University, Cardiff, CF10 3AT UK; 4 Department of Neurosurgery, Leeds General Infirmary, Leeds, UK

**Keywords:** Traumatic brain injury, Bifrontal contusions, Systematic review, Neurosurgical outcomes

## Abstract

Traumatic bifrontal contusions (TBC) form a recognised clinical entity among patients with traumatic brain injury (TBI). This study aims to systematically review current literature on demographics, management, and predictors of outcomes of patients with TBC. A multi-database literature search (PubMed, Cochrane, OVID Medline/Embase) was performed using PRISMA as a search strategy. Studies were selected by predefined selection criteria (PROSPERO: CRD42018055390), and risk of bias was assessed using an adapted form of ROBINS-I tool. Of the 275 studies yielded by the literature search, seven articles met the criteria for inclusion, all of which were level III evidence. Total cohort consisted of 468 patients; predominantly male (*n* = 5; 303/417 patients) with average age 44.3 years (range, 7–81). Falls (44.9%) and road traffic accidents (46.6%) were the commonest mechanisms of injury with an average presentation GCS of 9.2 (*n* = 3, 119 patients). GCS on admission of ≤ 13.1 and contusion volume at day 2 post-injury of ≥ 62.9cm^3^ were associated with increased risk of deterioration needing surgical interventions (*n* = 1, 7 patients). The majority of patients underwent surgery; the average GOS was 4, at an average follow-up duration of 11.7 months (*n* = 6, 356 patients). The currently available evidence on the management of TBC is scarce. Larger multicentre well-designed studies are needed to further delineate the factors behind acute deterioration, the effectiveness of management options. Once in place, this can be used to develop and test an algorithmic approach to management of TBC resulting in consistently improved outcomes.

## Introduction

**Fig. 1:**
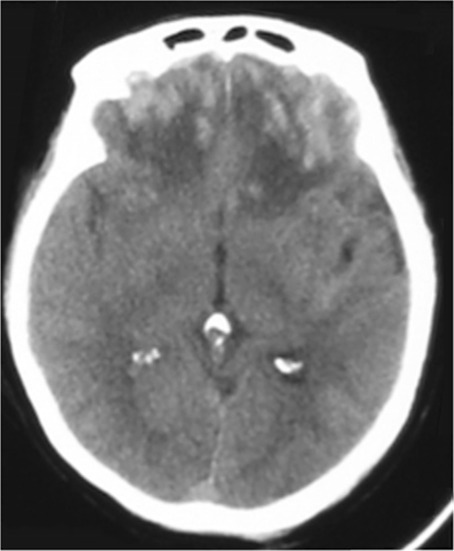
CT image of traumatic bifrontal contusions (adapted from Gao et al. [3])

Traumatic brain injury (TBI) is a leading cause of mortality and morbidity worldwide [6]. Various patterns of cerebral contusions are commonly seen after TBI. When involving both frontal lobes, such contusions may give rise to a particular subtype known as traumatic bifrontal contusions (TBC) (Fig. 1). TBC has been ominously associated with the “talk and die” paradigm due to its clinical characterisation [9]. Deterioration is thought to occur as a result of rostro-caudal displacement of the brain, culminating in central herniation [12]. Bilateral involvement of the frontal lobes means that early warning indicators in the form of lateralizing signs no longer manifest. Also, the epileptogenic nature of the basilofrontal lobe means that there is an increased risk of seizures and sudden deterioration [15]. Current treatment approaches are centred around the generic management of TBI: hyperosmolar therapy using hypertonic saline or mannitol to reduce cerebral oedema, correction of coagulation abnormalities, use of intracranial pressure (ICP) monitoring, and surgical intervention where necessary [10]. Whilst the role of decompressive craniectomy in the management of diffuse TBI has become increasingly unclear in light of recent trials [1], bifrontal decompressive craniectomy (BDC) remains a key surgical intervention for treatment of TBC. Other surgical procedures include bifrontal craniotomy (BC), with or without contusionectomy. However, clearer strategies for the management of TBC are required, to guide necessity for ICP monitoring, appropriate timing of surgical interventions, and indicators for prognosis in the longer term. In this study, we systematically reviewed the literature and evaluated the level of available evidence to guide management of TBC and identify predictors of their outcome.

## Methods

The structure of the search strategy for this literature review was based on the Preferred Reporting Items for Systematic Reviews and Meta-Analyses (PRISMA) guidelines [7]. The protocol for this systematic review is registered on PROSPERO (CRD42018055390).

### Search strategy

**Fig. 2:**
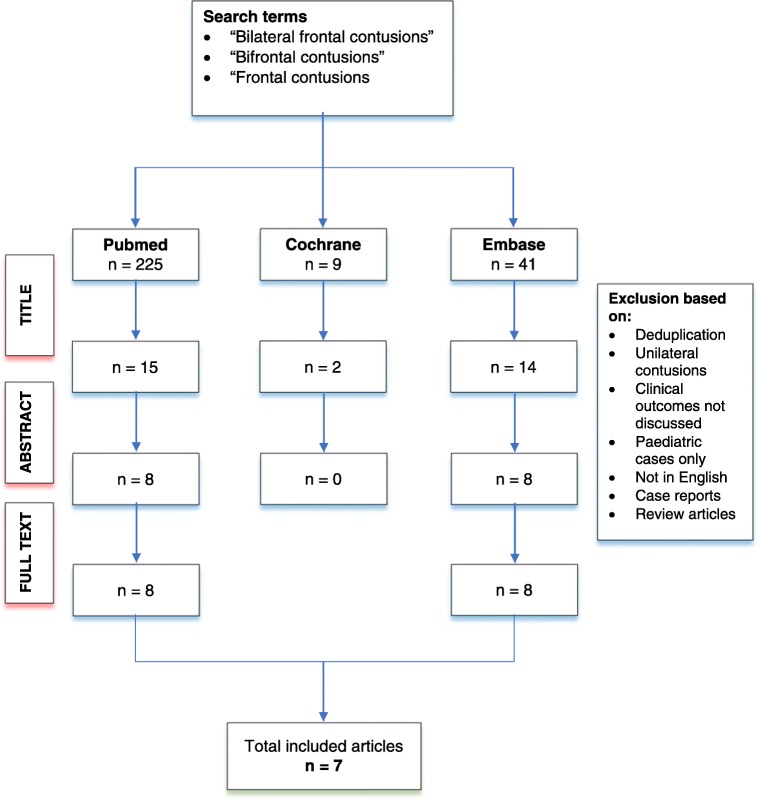
Flowchart depicting multi-database literature search for predictors of outcome in the management of TBC

A multi-database literature search (PubMed, Cochrane, and OVID Medline/Embase) was performed to identify relevant full text articles in English from January 1947 to January 2018 by authors NZ and SM (Fig. 2). Search terms included “bifrontal contusions”, “bilateral frontal contusions”, and “frontal contusions”. Difference in opinion on study inclusion was settled by consensus between authors NZ and SM, mediated by senior author MZ. The bibliographies of relevant articles were screened for further relevant citations.

### Study inclusion

All full text articles detailing the management and follow-up of patients with TBC were included. Specific attention was given to articles detailing GCS on admission, contusion volume, and demographic details. Studies of paediatric cohorts alone, case reports, review articles, commentaries, and non-English articles of TBC were excluded.

### Data analysis

All included studies were evaluated with respect to study design, patient cohort, mechanism of injury, clinical findings on admission, imaging parameters, management, and outcome. Outcome was assessed primarily according to longer-term follow up following discharge. Given the importance of sudden deterioration in TBC patients, we attempted to characterise the group of patients that deteriorated acutely with respect to reported parameters such as GCS and contusion volume, subsequent management, and final outcomes. Critical appraisal of included articles was performed by authors FS, MA, and DS, aided by use of an adapted version of the ROBINS-I tool to assess risk of bias [13]. Low number of included studies and heterogeneity of studies resulted in descriptive analyses being performed without meta-analyses.

## Results

### Study characteristics

A total of seven articles were included (see Table 1). All included articles were retrospective studies with a particular focus on TBC patients. Geographical locations included Scotland [12], USA [9], China [4, 16, 17], and India [11]. Sampling periods ranged from 1 to 14.5 years, with an average of 5.6 years.

**Table 1:** Characteristics of studies

	Author	Year	Location	Title	Design	*N*	Cohort	Outcome
1	Statham	1989	Scotland	Delayed deterioration in patients with traumatic frontal contusions	Retrospective	8	Patients with traumatic frontal contusions, without other major lesions or diffuse brain injury.	Assessed within 48 h of admission, at discharge, and GOS at 6 months
2	Petersen	2005–2010	USA	Talk and die revisited: bifrontal contusions and late deterioration	Retrospective	13	Patients with severe TBC only, defined by clinical/imaging criteria. Group with acute neurological deterioration compared with group that did not.	Modified Rankin score at 1 year.
3	Liang Gao	2003–2009	China	Intensive management and prognosis of 127 cases with traumatic bilateral frontal contusions	Retrospective	127	Patients with TBC and no other major lesions, defined by strict imaging criteria. ICP-monitored- and non-ICP groups compared for multiple variables.	GCS at discharge, mortality, GOS at 6 months.
4	Dong	2006–2009	China	Endoscopy-assisted cerebral falx incision via unilateral approach for treatment of dissymmetric bilateral frontal contusion	Retrospective	61	Comparing two surgical approaches: traditional BDC and endoscope-assisted unilateral cerebral falx incision.	GOS at 6-month post-injury.
5	Wu	2007–2012	China	The diagnosis and surgical treatment of central brain herniations caused by traumatic bifrontal contusions	Retrospective	63	Patients with TBC that were managed with BDC	GOS at a mean of 22 months with a range of 6–52 months
6	Sarma	2009–2014	India	Bifrontal contusions: what is the best surgical treatment?	Retrospective	98	Patients with TBC alone that were managed surgically.	In-hospital mortality rate, GOS at varying follow-up time points.
7	Zhaofeng	2000–2015	China	Surgical treatment of traumatic bifrontal contusions: when and how?	Retrospective	98	Patients with TBC alone managed with bifrontal DC.	GOS at 3 months.

### Patient cohort

A total of 468 patients were found across all included studies. Of studies specifying gender (*n* = 5), 72.7% (303/417) were male and 27.3% (114/417) were female. In studies reporting age (*n* = 5, 362 patients), average age of patients was 44.3 years (range 7–81). One study did not report age [12], and another study [11] only reported median age as 47 years. Patients were therefore predominantly male with an average age above 40 years.

### Mechanism and severity of injury

Mechanism of injury was reported by six studies, accounting for 459 patients. This consisted predominantly of falls (206 patients, 44.9%) and road traffic accidents (214 patients, 46.6%). Other causes included accidental injury (20 patients, 4.4%), assault (3 patients, 0.7%), and explosions (2 patients, 0.4%). The remaining 14 patients (3.1%) were reported as “Other”. One study [12] consisting of eight patients did not report mechanism of injury, and another study [9] did not report mechanism for one patient given that he/she was lost to follow-up.

GCS on admission was reported by all included studies (*n* = 7). Two studies reported GCS on an individual basis [9, 12]. One study [11] provided mean GCS on admission for a sample of 98 patients. The mean GCS on admission across these three studies (119 patients) was 9.4. The remaining four studies reported GCS on admission within differing ranges. Two studies [2, 4] reported 44 patients in the severe TBI category (GCS 3–5), 69 patients in the moderate TBI category (GCS 9–12), and 75 patients in the mild TBI category (GCS 13–15). Wu et al. (2014) [16] reported GCS pre-surgical intervention rather than on admission, with GCS 3–7 in 25 patients, GCS 8–12 in 33 patients, and GCS 13–15 in 5 patients. The remaining study [17] reported GCS 6–8 in 9 patients, GCS 9–12 in 38 patients, and GCS 13–15 in 51 patients.

### Imaging parameters

Not all studies provided strict imaging criteria for the diagnosis of TBC [2, 16] (see Table 2). One study [12] included patients depending on CT evidence of TBC, and further subdivided into limited and extensive subgroups, but no parameters were given for this diagnosis or distinction. Patients with any other major lesions were excluded, but parameters were not specified. Peterson et al. [9] defined their cohort as severe TBC based on specific contusion volume limits. In contrast to other studies, this definition was based on imaging performed at day 2 post-injury to account for blossoming of contusions. The conventional “AxBxC/2” method was used for calculating volume of contusion. The same method was used to measure surrounding hypodense perifocal oedema. Another study [4] specified imaging exclusion criteria for co-existing intracranial traumatic lesions other than TBC. Sarma et al. [11] reported a mean volume of bilateral contusions as 28 mL, and patients were distinguished with respect to symmetry/asymmetry of contusions, although threshold values for this distinction were not reported. The remaining study [17] reported CT on admission (98 patients) as “spotted” contusions in 17 patients, mean contusion volume of 15.1 mL in 73 patients, and 30.2 mL in 8 patients. Method for volume quantification of TBC was unreported in both studies. Whilst CT was the imaging modality of choice across all studies, one study [16] also performed MRI on five patients to study the structural process of central brain herniation more closely.

**Table 2:** Details of inclusion/exclusion criteria, medical management, and surgical interventions in each study

Study	Criteria	Medical	Surgical
Statham 1989	CT evidence of TBC- no specific parameters	Not specified	ICH evacuation in one patient
Peterson 2011	•GCS ≥ 10 on admission	Mannitol or 3% saline aiming for Na^+^ > 150 and Osm > 300	Bifrontal decompressive craniectomy
	•Total contusion volume > 30 cm^3^, and unilateral volume > 10 cm^3^ on CT day 2 post-injury		
	•No other intracranial traumatic lesions		
Gao 2013	•CT evidence of TBC- no specific parameters	Osmolar treatment- aiming for 300–320 Osm	Bifrontal decompressive craniectomy
	•Exclude patients with EDH > 30cm^3^, SDH > 10 mm thick, midline shift > 5 mm, or any other mass lesions > 20cm^3^	ICP monitoring in those with:	Bifrontal craniotomy
		•GCS < 8	Removal of contusion
		•GCS 9–12 and agitation requiring sedation	
		•CT signs of deterioration and GCS drop of > 2	tissue in both
Dong 2012	•CT evidence of TBC- no specific parameters	•ICP monitoring	Bifrontal decompressive craniectomy (if ICP > 25 mmHg after mannitol administration) Endoscope-assisted unilateral cerebral falx incision when: (i) unilateral frontal contusion with volume < 15 mL, (ii) angle of two frontal angulus of lateral ventricles more than 120° and effacement of basal cisterns, (iii) deteriorating consciousness with ICP > 25 mmHg
		•Mannitol	
Wu 2014	•CT evidence of TBC- no specific parameters	Only post-operative care specified:	Bifrontal decompressive craniectomy
	•Surgically managed by BDC	•Therapeutic temperature reduction	
		•ICP control	
		•Nutritional support	
		•Hyperbaric oxygen	
Sarma 2015	•CT evidence of TBC- no specific parameters	Hyperosmolar agents	Bifrontal decompressive craniectomy
	•No other intracranial traumatic lesions		Bifrontal craniotomy + contusion evacuation
			Unilateral contusion evacuation
	•Surgical management only		
Zhaofeng 2016	•CT evidence of TBC- no specific parameters	•Mannitol	Modified bifrontal decompressive craniectomy
		•Furosemide	
		•Anti-convulsant medications	
	•No evidence of multi-organ injury/dysfunction		
	•No other intracranial traumatic lesions		
	•GCS < 5		
	•Surgical management only		

### Management

Three key aspects of management were assessed: medical management, presence/absence of ICP monitoring, and surgical intervention. Except for one study [12], all studies specified medical therapies with varying levels of detail. Two studies [4, 9] described osmotic therapy based on biochemical targets. Others gave no plasma-based parameters as such. Wu et al. (2014) [16] only specified postoperative care, which entailed core temperature lowering strategies, ICP control, nutritional support, and hyperbaric oxygen therapy. The remaining studies provided information on the use of hyperosmolar agents without further distinction in one study [11] and use of mannitol, furosemide, and anti-convulsant medications in another [17].

Use of ICP monitoring was evaluated directly in three studies [4, 9] [2]. In one study [9], ICP monitoring was performed in four patients. All four had a GCS greater than 11, and two deteriorated acutely. The relationship between use of ICP monitoring and outcome, and criteria for employing ICP monitoring were not discussed. Gao et al. (2013) [4] emphasised the use of ICP monitoring and its relationship to various outcome measures. Of the 127 patients, 39 underwent ICP monitoring. Criteria for ICP monitoring were defined as (i) GCS < 8, (ii) GCS 9–12 and agitation necessitating sedative medications, or (iii) CT evidence of deterioration and drop in GCS of more than two points. The use of monitoring was analysed in relation to duration of ICU stay, duration of hospitalisation, and GOS at 6 months (discussed below). ICP monitoring was performed with an intraventricular probe in this study. In the remaining study [2], ICP monitoring was used in all patients, and decision for emergency BDC was made based on persistently increased ICP > 25 mmHg despite mannitol administration.

The majority of patients across the included studies underwent surgery (329 patients, 80.8%). Various surgical interventions were described. Three studies only considered patients undergoing surgery (12–14). Of those, one study [16] utilised BDC with removal of frontal contusions and haemorrhaging tissue as their sole surgical intervention. Another [11] determined their surgical approach in accordance with TBC morphology. Depending on whether or not contusions were symmetrical, one of the three surgical procedures was employed: BDC alone, BC with evacuation of contusions, and unilateral evacuation of frontal contusion. Finally, Zhaofeng et al. (2016) [17] used a modified BDC approach in all patients. The remaining three studies evaluated all patients with TBC, with only a proportion operated on. In one study [12], one patient underwent unilateral evacuation of frontal contusion while the remaining seven patients were managed conservatively. In another study [9], BDC was only performed on patients that deteriorated acutely (7 out of 13). BDC was performed on six patients whilst the seventh did not have surgery due to family refusal. The procedure involved BDC without evacuating contusions, and cranioplasty was performed at 3 months. Gao et al. (2013) [4] divided their surgical cohort into ICP- and non-ICP-monitored subgroups. In total, 63 of the 127 patients underwent surgical interventions, which included BC (15 out of 63) and BDC (41 out of 63) as well as insertion of ICP monitor without further surgery (7 out of 63). Both surgical procedures included removal of frontal contusion tissue and haematoma. A different approach for managing unilateral dominant TBC based on specific imaging criteria was described in one study (31 patients, see Table 2), involving endoscopy-assisted unilateral cerebral falx incision (UCFI) [2].

### Deterioration

Three studies [4, 9, 12] evaluated the clinical trajectory of deterioration in TBC patients in particular. Statham et al. [12] observed deterioration in two patients, both of whom had extensive TBC. The other patient was GCS 5 on admission and improved in the first 2 days to GCS 9, but deteriorated on day 9 and died. Peterson et al. [9] defined parameters for acute deterioration, which included (i) drop in GCS of more than four points or (ii) persistent ICP > 20 (units not provided) despite maximal medical therapy in those with ICP monitors. Of the 13 included patients, seven patients (53.8%) deteriorated acutely, two of whom had ICP monitors. For patients that deteriorated, on average: (i) GCS on admission was 13.1, (ii) contusion volume at day 2 post-injury was 62.9cm^3^, (iii) deterioration took place on day 4.4 post injury, and (iv) oedema volume at time of deterioration was 69.7cm^3^. The third study [4] reported day of deterioration in ten patients and subsequent outcomes at 6 months. Of these patients, five underwent ICP monitoring. On average across all studies (19 patients), deterioration occurred on day 4 and GCS on admission was 12.

### Outcomes

A total of 368 patients (75.4%) across all studies were followed up, and 100 patients (24.6%) had no follow-up data (see Table 3). Peterson et al. [9] lost one patient (1/13, 7.7%) to follow-up and Sarma et al. (2015) [11] lost 20 patients (20/98, 20.4%) to follow-up. One study [4] had two sources of missing data: (i) 15 patients (11.8%) were lost to follow-up, and (ii) follow-up data on 64 patients (50.4%) from the conservative management group were not consistently reported (see the “Discussion” section). Average duration of follow-up across all studies (*n* = 7, 368 patients) was 13 months. Except one study [9], all included studies measured GOS at various time points. Average GOS across these studies was 4, at an average follow-up duration of 11.7 months (*n* = 6, 356 patients). Peterson et al. 2011 [9] measured modified Rankin score at 1-year follow-up, with an average score of 2.3 (12 patients). A total of 45 patients died, accounting for 9.6% of patients across all studies.

**Table 3:** Outcomes and follow-up of TBC patients in included studies

Study	Follow up (no., % of cohort)	Mean duration (months)	Scoring system	Average score	Deaths (no., % of cohort)
Statham 1989	8, 100	6	GOS	2	1, 12.5
Peterson 2011	12, 92.3	52	Modified Rankins	2.3	2, 15.4
Gao 2013*	48, 37.8	6	GOS	3.9	–
Dong 2012	61, 100	6	GOS	4.2	2, 3.3
Wu 2014	63, 100	22	GOS	4.2	2, 3.2
Sarma 2015	78, 79.6	23	GOS	2.7	36, 36.7
Zhaofeng 2016	98, 100	3	GOS	4.7	2, 2.0

*Please see text for a discussion of follow-up in this study

### Risk of Bias

Included studies were assessed using the ROBINS-I tool [13] with respect to the following categories: confounding, selection, intervention classification, deviation from intervention, missing data, measurement of outcome, and selection of reported result (see Table 4).

**Table 4:** Demonstrates use of ROBINS-I tool for assessment of risk of bias in included studies

	Study	Confounding	Selection	Intervention classification	Deviation from intervention	Missing data	Measurement of outcome	Selection of reported result	Overall
1	Statham 1989	Low	Moderate	Low	Low	Moderate	Low	Low	Moderate
2	Peterson 2011	Moderate	Moderate	Low	Moderate	Moderate	Low	Low	Moderate
3	Gao 2013	Moderate	Moderate	Moderate	Low	Critical	Low	Critical	Critical
4	Dong 2012	Moderate	Serious	Low	Low	Low	Low	Low	Serious
5	Wu 2014	Critical	Serious	Low	Low	Low	Serious	Low	Critical
6	Sarma 2015	Critical	Low	Serious	Low	Moderate	Serious	Critical	Critical
7	Zhaofeng 2016	Critical	Moderate	Low	Low	Low	Low	Low	Critical

## Discussion

Despite the clinical conundrum that TBC poses, it is clear that there is a paucity of evidence on prognostic indicators of deterioration, utility of ICP monitoring, and effective management strategies (see Table 5). This may have been accentuated by the stringency of our inclusion criteria. Clinically pertinent details such as the use of anticoagulants remain to be clarified, as is the case for TBI in general [5, 8]. The majority of patients included across all studies were male (69.8%), and an average age of above 40 years. Whilst this is consistent with the demographics of TBI [3], gender and age specific differences should be explored. Only one study [4] evaluated outcome at 6 months in age-based subgroups. Statistical tests were not performed, precluding any quantitative comparisons. Also, reports of outcome were inconsistent within this study. Despite reporting that 15 patients were lost to follow-up within the surgical cohort, GOS scores are reported for all patients when discussed with respect to age. Due to this inconsistency, outcomes for the surgical cohort alone were included in our analyses, as this was discussed extensively in comparisons between ICP and non-ICP subgroups. Remaining patients in the conservative group were excluded from outcome analysis to avoid error.

**Table 5:** Summary of discussion points

	Statham 1989	Peterson 2011	Liang Gao 2013	Dong 2012	Wu 2014	Sarma 2015	Zhaofeng 2016
Demographics	–	–	+	+	+	+	+
Mechanism of injury	–	+	+	+	+	+	+
Strict imaging criteria	–	+	–	–	–	–	+
GCS on admission	+	+	+	+	–	+	+
Clinical trajectories	+	+	+	–	–	–	–
Role of ICP monitoring	–	–	+	+	–	–	–
Role of operative intervention	+	+	+	+	+	+	+
Outcomes	+	+	+	+	+	+	+

The earliest included study [12] performed a retrospective review of patients with frontal contusions presenting to their unit over the course of a year. A total of 20 patients were included initially, of which two patients were excluded due to unavailability for follow-up. The TBC group consisted of eight patients, and was further divided into limited and extensive subgroups based on the depth of contusions. Of all patients, two showed deterioration, both of whom had extensive bilateral contusions: one was successfully managed with contusionectomy, while the other died. This study provides a broad overview of a consecutive number of patients with isolated TBC, presenting their outcomes at 6 months as well as detailing clinical trajectories for two cases. However, several details are left unspecified including (i) demographics, (ii) presence/absence of ICP monitoring (except for one case), (iii) exact imaging criteria for diagnosis of bifrontal contusions, and (iv) comparison of surgical and conservative management.

The subsequent study [9] was performed approximately two decades later. TBC patients were retrospectively evaluated over a 5-year period, with data collected from a prospective database. More refined inclusion criteria were chosen: (i) only severe TBC, defined as total bilateral contusion volume of > 30cm^3^ and minimum contusion volume of 10cm^3^ unilaterally, (ii) GCS ≥ 10 on admission, and (iii) no evidence of other intracranial traumatic lesions. Contusion volumes used to screen for inclusion were measured on day 2. Authors chose this time point to account for “blossoming” of contusions from initial admission. Initial contusions “blossomed” in nine patients (69%) by day 2. Only six of the nine patients experienced acute deterioration at some stage post admission within the first week, but this did not necessarily go hand-in-hand with radiological progression. It follows that the relationship between clinical and imaging parameters warrants further study. The shortcomings of this study include (i) rationale behind the decision for ICP monitoring was not given, (ii) inclusion criteria state GCS ≥ 11 on admission, yet subsequent discussion reveals two patients presenting with GCS 10, and one patient presenting 3-day post-injury with GCS 3, and (iv) despite investigating several relevant parameters, the study prevents any quantitative evaluation of deteriorating and non-deteriorating subgroups due to the small sample size and lack of statistical comparison.

Across all studies, BDC and BC were the predominant surgical approaches reported. One study [2] compared UCFI and BDC with respect to GOS at 6 months and other secondary outcome measures. There was no significant difference outcome, but UCFI was associated with shorter operative time and duration of hospital stay. However, UCFI was only performed under specific conditions (see Table 2), precluding meaningful comparison with BDC. Future studies comparing both approaches for unilateral dominant TBC are required to establish the validity of UCFI. Clinical deterioration was not discussed, and radiological parameters for TBC were not reported. ICP monitoring was performed in all patients and used to guide timing of surgical intervention.

The remaining included studies were published over the subsequent few years. Gao et al. [4] collected retrospective data on 127 TBC patients over a 5-year period. The following variables were investigated: (i) severity of TBI, (ii) use of ICP monitoring, (iii) type of surgical intervention, (iv) biochemical markers, and (v) outcomes. Less stringent inclusion criteria were employed, allowing other traumatic intracranial lesions within certain size limits. Thus, whilst a more pragmatic approach is adopted to accommodate the complexity of TBI, TBC is not evaluated in isolation. Additionally, no strict radiological definition of TBC was given, precluding severity-based evaluation. Due to a large sample size, individual clinical trajectories could not be followed in the same fashion as in Peterson et al. [9]. Surgically and medically managed groups were identified. Within the surgical group, ICP and non-ICP subgroups were recognised. Furthermore, the ICP and non-ICP subgroups were compared for various parameters, demonstrating significantly shorter duration of ICU (*p* = 0.013) and overall hospital stay (*p* = 0.001), as well as fewer days of osmotic therapy (*p* = 0.008) for patients belonging to the ICP subgroup. Interestingly, of surgical patients followed up at 6 months, the ICP cohort also had a significantly higher GOS at 6 months compared to non-ICP cohort (*p* = 0.025). However, the indications for ICP monitoring in this study were not clearly defined. Superior outcomes in the ICP group suggest a potentially useful role in TBC management, although further evidence is required. Despite multiple data points, the effects of only a few of these on GOS scores were calculated: age, progression to surgery, and ICP monitoring. Only the latter was accompanied by statistical comparisons. Similar to Peterson et al. [9], deterioration was defined in a specific manner by the presence of one of the following parameters: (i) decrease of more than two points on GCS, (ii) persistently elevated ICP > 25 mmHg despite medical management, or (iii) CT changes showing significant increase in contusion volume, but this was not clearly defined. In summary, this study builds on previous work in terms of defining severity of TBI, evaluating the use of ICP, and providing an algorithmic approach to management. However, further characterisation of the relationship between presentations, interventions, and outcomes is required, for identifying prognostic indicators of TBC.

Wu et al. [16] performed a retrospective study of TBC patients that had been managed with BDC over a 5-year period. Of included patients, approximately half underwent emergency surgery on admission, whilst the remaining patients had surgery after a period of observation. Imaging criteria for definition of TBC were not provided. Of the 63 patients investigated, seven died, and the remainder were followed up for a mean duration of 22 months. Compared to previous studies, there are multiple drawbacks: (i) GCS on admission was not provided; only pre-operative GCS was reported; (ii) the presence of other traumatic lesions was not a part of the exclusion criteria; (iii) GOS was not performed at consistent time points across all individuals; (iv) a definition for TBC was not provided; and (v) duration of observation periods were not reported, precluding any assessment of aspects of deterioration.

Similarly, another retrospective study [11] evaluated 98 TBC patients that had been surgically managed over a 5-year period. As discussed above, one of three surgical interventions was carried out in each case, depending on the symmetry of TBC. Despite being the only study to discuss this quality of TBC, it did not set any parameters to allow objective stratification. Nonetheless, given that current understanding of acute deterioration revolves around the antero-posterior displacement of the brain [16], this variable may be of interest and deserves more attention in future studies. Whilst demographics, GCS on admission, and average contusion volume were recorded, their relationship with outcome was not fully assessed. Although the effect of individual surgical procedures on in-hospital mortality rate was studied (55% in the BDC group, 35.3% in the BC group, and 8.3% in the unilateral contusion evacuation group), only the overall effect of surgical intervention on GOS at a range of time points was reported.

The most recent study [17] retrospectively analysed a cohort of TBC patients managed with BDC over a 15-year period, with a sample size of 98 patients. TBC alone was studied, and patients with other traumatic intracranial lesions or evidence of multi-organ dysfunction were excluded. Patient demographics along with contusion volumes were calculated. In contrast to previous studies, an operation-timing score was developed to determine the necessity for surgical intervention. This score was centred primarily around (i) time to deterioration and (ii) extent of deterioration based on either GCS or CT findings. A steeper deterioration was required at earlier periods post-injury to affirm the decision for surgery. Although an average operation timing of 4.5 days post admission is provided, specific details of deterioration are not provided, meaning that individual clinical trajectories cannot be assessed. Although GOS at 3 months was measured, its association with different parameters was not studied. Therefore, the identification of predictors of outcome is difficult. Despite its drawbacks, the introduction of the operation-timing score in this study could be evaluated further in future. Indeed, authors chose to use GCS and imaging criteria without ICP monitoring to avoid associated risks such as haemorrhage and infection [14], and demonstrated successful results. This is in contrast to conclusions from Gao et al. [4], where the utility of ICP monitoring is emphasised.

**Fig. 3:**
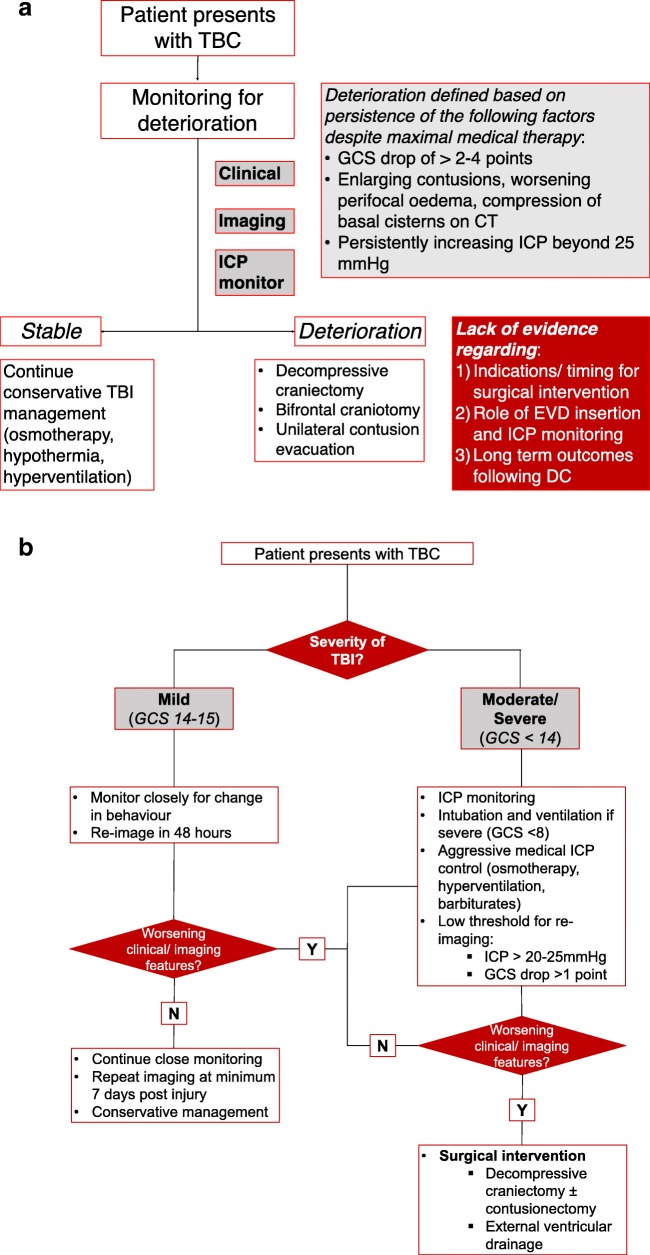
**a** Algorithmic approach to management of TBC based on included studies (level 4 evidence), further studies are required to clarify (i) definitive clinical and imaging parameters for deterioration, (ii) the role of ICP monitoring, and (iii) indications for neurosurgical intervention and optimal approach. **b** Proposed approach to management of TBC based on authors’ experience

In conclusion, there is currently a lack of evidence on many aspects of the management of TBC. The stringency of our inclusion criteria further limited widespread appraisal of evidence. However, based on available evidence, some consistent themes emerge (Fig. 3): (i) initial stratification of patients based on TBI severity should guide decisions on ICP monitoring and medical management of ICP; (ii) a low threshold for re-imaging based on clinical deterioration (GCS drop > 1 point) or increasing ICP (beyond > 25 mmHg or acutely increasing); (iii) routine imaging at 48 h and 7 days independent of clinical progression; and (iv) urgent surgical intervention in high risk patients with severe TBI and features of deterioration on clinical or imaging parameters. Future studies must (i) systematically investigate the factors behind acute deterioration, with the aim to correlate clinical and imaging parameters as well as their respective trajectories with the risk of deterioration, (ii) clarify the role of ICP monitoring as a prognostic indicator, and (iii) use this information to develop and test an algorithmic approach to management of TBC resulting in consistently improved outcomes.
